# Oxidative Stress and Inflammation-Related mRNAs Are Elevated in Serum of a Finnish Wet AMD Cohort

**DOI:** 10.1167/iovs.65.13.30

**Published:** 2024-11-15

**Authors:** Mikko Liukkonen, Hanna Heloterä, Leea Siintamo, Bishwa Ghimire, Pirkko Mattila, Niko Kivinen, Joanna Kostanek, Cezary Watala, Maria Hytti, Juha Hyttinen, Ali Koskela, Janusz Blasiak, Kai Kaarniranta

**Affiliations:** 1 Department of Ophthalmology, University of Eastern Finland, Kuopio, Finland; 2 Department of Ophthalmology, Kuopio University Hospital, Kuopio, Finland; 3 Institute for Molecular Medicine Finland, University of Helsinki, Helsinki, Finland; 4 MediCity Research Laboratory, University of Turku, Turku, Finland; 5 Department of Haemostatic Disorders, Medical University of Lodz, Lodz, Poland; 6 Faculty of Medicine, Mazovian Academy in Plock, Plock, Poland; 7 Department of Molecular Genetics, University of Lodz, Lodz, Poland

**Keywords:** cardiovascular disease, DESeq2, fgsea, neovascular AMD, RNA-seq, ROS, wet AMD

## Abstract

**Purpose:**

Localized diseases can be affected by and affect the systemic environment via blood circulation. In this study, we explored the differences in circulating serum mRNAs between patients with wet AMD (wAMD) and controls.

**Methods:**

Blood samples were obtained from 60 Finnish patients with wAMD and 64 controls. After serum preparation and RNA sequencing, the count data was examined for differentially expressed genes (DEGs) and further checked for enriched molecular pathways and ontology terms as well as links to clinical data.

**Results:**

We found many DEGs and some enriched pathways, including the inflammation and cell survival-associated pathway tumour necrosis factor alpha (TNF-α) signaling via nuclear factor kappa–light-chain-enhancer of activated B cells (NF-κB). The related DEGs were oxidized low-density lipoprotein receptor 1 (*OLR1*), salt inducible kinase 1 (*SIK1*), and coagulation factor III (*F3*). DEGs from degradative macular and retinal processes were also examined, many of which were also related to cardiovascular disease and maintenance. Additionally, DEG counts were inspected in relation to clinical and anti-VEGF treatment parameters, and glutamine amidotransferase-like class 1 domain-containing 3A (*GATD3A*) levels were found to be significantly lower in patients with wAMD treated with anti-VEGF.

**Conclusions:**

Differentially expressed systemic mRNAs that are linked to mitochondrial function, oxidative stress, and inflammation may have a role in the pathology of wAMD. Our observations provide new data for the understanding of the progression of wAMD.

AMD is one of the most common causes of vision impairment in the elderly both worldwide and in Finland.^1^^,^^2^ It causes detrimental changes in the central vision, hampering and eventually preventing activities that require clear eyesight, such as reading and driving. AMD first manifests as early stage AMD with small drusen formation and minor pigmentary changes that do not impact vision.^3^ It can then gradually progress into the more serious dry (geographic atrophy) or wet (neovascular) AMD (wAMD). Medications for slowing down the progression of geographic atrophy have only recently received U.S. Food and Drug Administration approvals, whereas wAMD has had medications available for two decades.^4^^,^^5^ Treatment of wAMD typically aims to mitigate the formation of invasive leaky blood vessels from the choroid into the retina, called choroidal neovascularization (CNV).^6^ The most common treatments are anti-VEGF injections, which aim to slow down CNV and maintain normal blood vessel function.

The main cause of AMD is aging, but mutations in some genes have been shown to predispose to AMD, including the complement factor H (*CFH*), age-related maculopathy susceptibility 2 (*ARMS2*), and HtrA serine peptidase 1 (*HTRA1*) genes.^7^ Additionally, lifestyle factors such as smoking, excess weight, and cardiovascular diseases are often considered to increase the chance of developing AMD.^8^^,^^9^

AMD progression has been shown to involve many kinds of altered cellular processes in the retinal pigment epithelium (RPE). These include impaired mitochondrial function, dysfunctional autophagy, increased reactive oxygen species (ROS) production, inflammation, and fibrosis.^10^^–^^16^ RPE cells are mostly dormant and do not get renewed, even as damage accumulates due to age and issues in the above-mentioned cellular processes, leading to more pronounced deterioration of vision.^17^

Given that the cellular processes involved in AMD (inflammation, angiogenesis, etc.) are not specific to the eye, it can be speculated that systemic signaling may play a role in AMD progression.^18^^–^^20^ Conversely, treatment of AMD can have a detectable effect within the systemic circulation, but the effects vary between different medications.^21^^,^^22^ Additionally, at this time, it is difficult to tell which, if any, blood biomarkers will prove relevant in monitoring AMD progression; studies have found a multitude of potential biomarkers, but the findings have often turned out to be difficult to replicate or even contradictory.^23^

There have been many studies inspecting the upregulation and downregulation of RNAs in AMD, from the single cell and tissue levels to aqueous humor and systemic circulation.^24^^–^^28^ These have concentrated mostly on the noncoding RNAs, miRNAs in particular, because those often have a regulatory effect on cellular functions. In recent years, circulating mRNAs have emerged as potentially useful biomarkers in recognizing and following disease progression.^29^^–^^31^ Finding and establishing circulating mRNAs that are significantly upregulated or downregulated in disease cohorts can help to understand underlying mechanisms that affect or are affected by the disease, although any findings will need to be ascertained through further studies to rule out stochastic effects.

As the mechanisms of AMD pathogenesis are still poorly known, studies on clinically useful biomarkers are justified. Study of the molecular mechanisms of AMD pathogenesis may answer the questions of why some AMD patients’ vision deteriorates faster, or why they react worse to treatment than expected. In this study, mRNA from the blood serum of Finnish patients with wAMD and control cohorts was sequenced and analyzed for differentially expressed genes (DEGs). The resulting DEGs were inspected for links to enriched molecular pathways and ontology terms, as well as clinical data.

## Methods

### Patient Blood Sample and Data Collection

This study is part of a larger clinical trial that has been listed in the EU Clinical Trials Registry (EudraCT Number: 2012-000765-20). The study was conducted in accordance with the Declaration of Helsinki and approved by the Ethics Committee of the Kuopio University Hospital (approval number 42/2014). Patient data were collected and stored in accordance with the new European Union's Regulation 2016/679 on the protection of personal data. Written informed consent was obtained from all patients involved in the study. Patients were asked to sign their approval for blood sample collection.

Blood samples from 60 patients with wAMD (mean age, 79.2 years; range, 58–96 years; male/female = 18/42) and 64 controls (cataract surgery patients without signs of retinal degeneration; mean age, 76.0 years; range, 57–93 years; male/female = 21/43) were gathered from patients at the Kuopio University Hospital. The treatment regimens of the patients with wAMD started with three once-a-month intraocular anti-VEGF injections, after which the treatment response was assessed. A new treatment series started either immediately or after a period of rest to allow the eye to recover, depending on the visual acuity and optical coherence tomography scan results. When visual acuity decreased and the patients no longer responded to treatment, the treatment ended. In some cases, additional singular anti-VEGF injections might also be assigned. For the patients with wAMD in our study, the median length of treatment was 3 years, with the treatment lengths ranging between 1 and 8 years. Possible reasons for the cessation of the gathering of treatment data on a patient included end of treatment due to lack of treatment response, patient death, patient choosing to not continue treatment, patient moving to a different health care region, patient getting potential confounding retinal diseases such as diabetic retinopathy, and other reasons. The most commonly applied anti-VEGF agent for our treatments was bevacizumab, followed by aflibercept.

**Figure 1.**
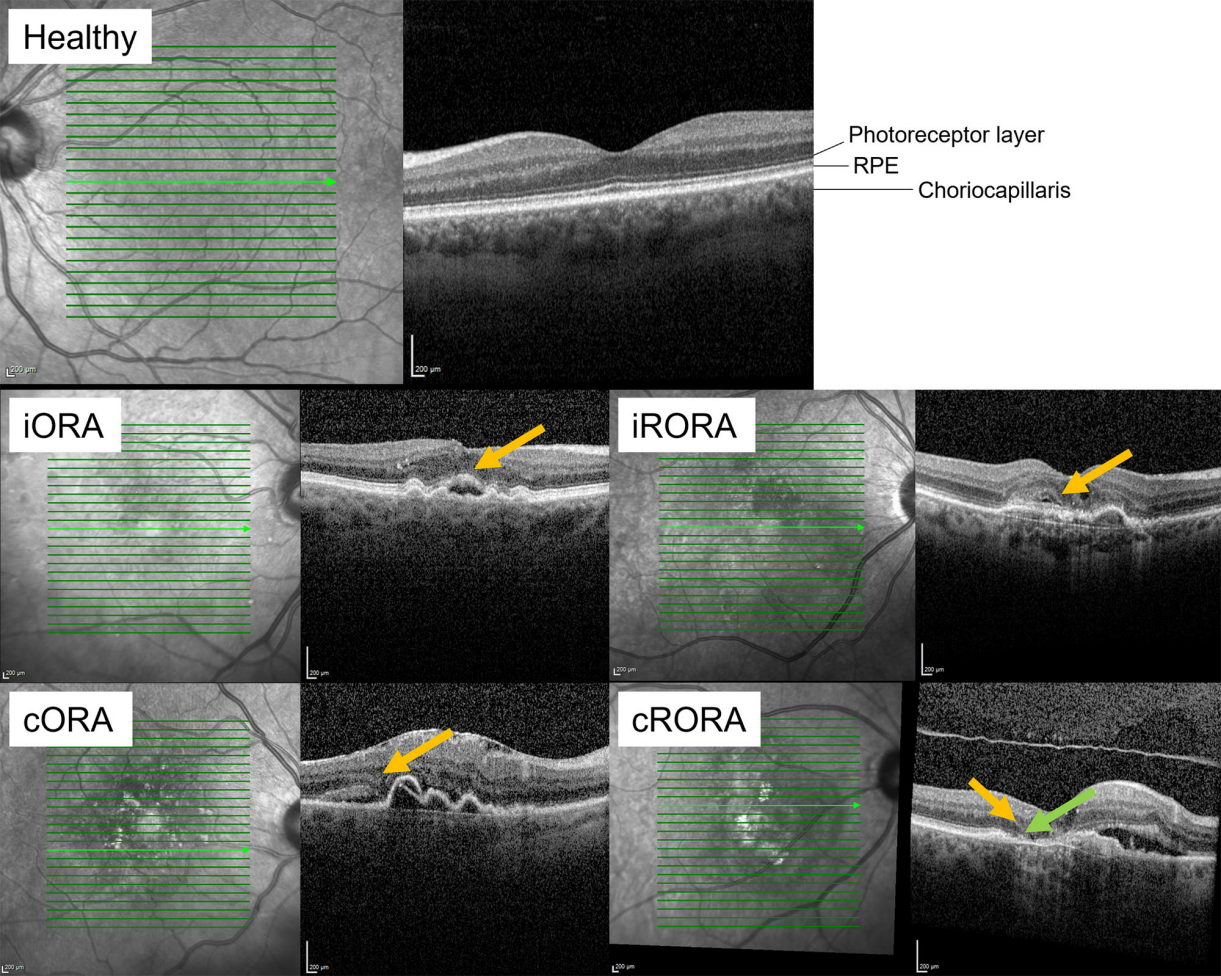
Optical coherence tomography images of healthy to severely atrophied retinas. Yellow arrows indicate outer retina damage and the thick green arrow indicates RPE level changes. cORA, complete outer retinal atrophy; iRORA, incomplete RPE and outer retinal atrophy; RPE, retinal pigment epithelium.

Blood serums were immediately centrifugally separated as previously described and stored at −70°C for up to 7 years, with 95% of the samples having been stored for 1–3 years before sequencing.^32^ The patients’ general characteristics and retinal properties were also acquired as previously described. In short, for appraising retinal atrophy, spectral domain optical coherence tomography (SPECTRALIS OCT2, Heidelberg Engineering, Heidelberg, Germany) imaging was used to capture the macular region, covering the fovea and part of the surrounding extrafoveal regions. Postinjection images closest to 1 full year from the beginning of treatment represented each respective treatment year (year 1, year 2, etc.) for gauging the patients’ retinal atrophy levels. Retinal atrophies were classified into four levels based on the amount of damage to the outer retina and RPE, examples of which can be seen in Figure 1.^33^ In addition, atrophies related to a torn RPE were only classified as such.

### RNA Extraction and Sequencing

RNA extraction was performed at the HiPREP Core at the Institute for Molecular Medicine Finland (FIMM) Technology Centre and sequenced at the FIMM Genomics NGS Sequencing unit at the University of Helsinki, supported by the Helsinki Institute of Life Science and Biocenter Finland. Total RNA was extracted from the serum samples (1 mL) using Maxwell RSC 48 Instrument and Maxwell RSC miRNA Plasma and Serum Kit (Promega, Madison, WI) according to the manufacturer's instructions, including a DNase treatment. RNA was eluted in 50 µL RNase-free water (35–40 µL recovered). Sample quality and quantity were determined using Agilent Bioanalyzer 2100 with the RNA 6000 Pico Kit (Agilent Technologies, Santa Clara, CA).

Library preparation from 800 ng of total RNA was performed according to the Illumina Stranded Total RNA with Ribo-Zero Plus reference guide (Illumina, San Diego, CA). Library quality check was performed using LabChip GX Touch HT High Sensitivity assay (Perkin Elmer, Shelton, CT) and libraries were pooled based on the concentrations acquired from the assay. The library pools were quantified for sequencing in a qPCR run using the KAPA Library Quantification Kit (KAPA Biosystems, Wilmington, MA). Sequencing was performed with the Illumina NovaSeq 6000 system using an S4 flow cell with a lane divider (Illumina, San Diego, CA). Sequencing data yield was good within sample type constraints. The read length for the paired-end run was 2 × 151 bp. The RNA sequencing datasets were quality checked and pre-analyzed as per the FIMM-RNAseq 2.0.7 workflow.^34^ The data discussed in this publication have been deposited in NCBI's Gene Expression Omnibus (GEO) and are accessible through GEO Series accession number GSE273435 (https://www.ncbi.nlm.nih.gov/geo/query/acc.cgi?acc=GSE273435).^35^

### Quantitative PCR

RNA was extracted from serum samples (*n* = 16; 8 per patient group) using the NucleoSpin miRNA Plasma Mini kit (Macherey-Nagel, Düren, Germany) and quantified using a NanoDrop Microvolume spectrophotometer (Thermo Fisher Scientific, Waltham, MA). Between 100 ng and 400 ng of RNA were reverse transcribed using the SuperScript IV Vilo Master Mix (Thermo Fisher Scientific). The resulting cDNA was analyzed using PowerUp SYBR Green Master Mix (Thermo Fisher Scientific) on a Quantstudio 5 Real-Time PCR system (Thermo Fisher Scientific) to determine RNA expression levels using the 2^−^^ΔΔCt^ method.^36^ The relative expression levels of salt inducible kinase 1 (*SIK1*) and glutamine amidotransferase-like class 1 domain-containing 3A (*GATD3A*) were measured and normalized to the levels of the housekeeping gene glyceraldehyde-3-phosphate dehydrogenase (*GAPDH*). The primer sequences used are presented in Table 1.

**Table 1.** Primer Pairs Used for qPCR

Gene	Forward 5′-3′	Reverse 5′-3′
*SIK1*	ACC AGG TTA TGG AAA CAA AGG A	CGA CAG GAT TTG CCA GAA CT
*GATD3A*	TGT CTG GAT GCG GAG TCT AC	TGG TCA ATC ACG TGC ATC TG
*GAPDH*	ACA ACT TTG GTA TCG TGG AAG G	GCC ATC ACG CCA CAG TTT C

*GAPDH*, glyceraldehyde-3-phosphate dehydrogenase; *GATD3A*, glutamine amidotransferase-like class 1 domain-containing 3A; *SIK1*, salt inducible kinase 1.

### Data Processing and Statistical Analysis

The acquired count data was filtered to protein-coding RNAs using data sourced from biomaRt 2.50.3, which were then filtered in R Statistical Software 4.1.3 with DESeq2 1.34.0 to include a minimum of 10 reads in at least seven patients when assessing the entire dataset.^37^^–^^40^ The DESeq2 analysis was then run according to package instructions and the resulting DEGs with alpha set to 0.05 were saved. The data were visualized using PCAtools 2.6.0 after variance stabilizing transformation, and the results were visualized using EnhancedVolcano 1.12.0 with and without apeglm log_2_-fold change (LFC) shrinkage.^41^^–^^43^

The data were further analyzed using the fgsea 1.20.0 (fast gene set enrichment analysis) package using the multilevel as well as 1,000,000 permutation analyses with otherwise default settings.^44^ Hallmark, curated, and ontology gene sets (H, C2, and C5) from the Molecular Signatures Database were used via the msigdbr 7.5.1 package.^45^^–^^47^ The inflammation-related Hallmark results were inspected further to identify the related DEGs. Benjamini–Hochberg method false discovery rate adjusted *P* values of 0.050 or less were considered significant for both types of analyses.^48^ To seek out significant differences in gene counts on a per-gene basis based on patient characteristics, such as retinal atrophy level and whether the samples had been gathered from treatment-naïve or treated patients, edgeR 3.36.0 was used to generate library size normalized counts per million data.^49^

Statistical calculations for the patient characteristics were done using SPSS Statistics 27.0.1.0 (IBM, Armonk, NY). To compare patient ages at sampling, the independent samples *t*-test was used, whereas the Mann–Whitney *U* test was used for BMI comparisons due to the non-normal distribution of values, and chi-square tests were used for gender, smoking, and medication comparisons. For the calculation of per-gene differences using patient characteristics, the counts per million data distributions between the selected groupings were compared using the Mann–Whitney *U* test.

The qPCR data was probed for statistically significant differences in GraphPad Prism 10.2.2 (GraphPad Software, Boston, MA). Pairwise comparisons were performed using the Mann–Whitney *U* test and a *P* value of less than or equal to 0.050 was considered statistically significant.

## Results

### Patient Characteristics

The basic information on the patients in this study is shown in Table 2. There was no significant difference in gender or smoking habits between the patients with wAMD and the controls, whereas the patients with wAMD had significantly higher age at sampling (*P* = 0.040) and body mass index values (*P* < 0.001). The patients with wAMD also had significantly more cases with blood pressure (*P* = 0.002) and anticoagulation (*P* = 0.004) medication usage.

**Table 2.** Patient Characteristics

Characteristics	wAMD (*n* = 60)	Control (*n* = 64)	*P* Value
Basic			
Age at sampling, years	79.2 ± 9.4	76.0 ± 7.2	0.040
Gender (M/F)	18/42	21/43	0.736
Smoking habits (no/random/active smoking)	42/15/3	41/14/9	0.232
BMI^*^	27.2 [20.2–36.6]	24.9 [19.6–49.4]	<0.001
Medication			
Blood pressure	52	40	0.002
Anti-cholesterol	27	29	0.972
Anticoagulation	15	4	0.004
Anti-aggregation	22	21	0.652
Retinal atrophy location			
Foveal	52		
Extrafoveal	8		
Retinal atrophy type			
iORA	11		
cORA	12		
iRORA	12		
cRORA	17		
RPE tear	6		
Unknown	2		
No. of samples in relation to the beginning of treatment			
Before treatment (treatment-naïve)	23		
After beginning of treatment (treated)	37		
Length of treatment, years	2.3 ± 1.8		

The basic and medication characteristics are baseline data, excluding age at sampling.

Values are mean ± SD, number of users/patients, or median [min–max].

* One missing value in the wAMD group.

BMI, body mass index; cORA, complete outer retinal atrophy; iRORA, incomplete RPE and outer retinal atrophy.

### RNA Data Characteristics

**Figure 2.**
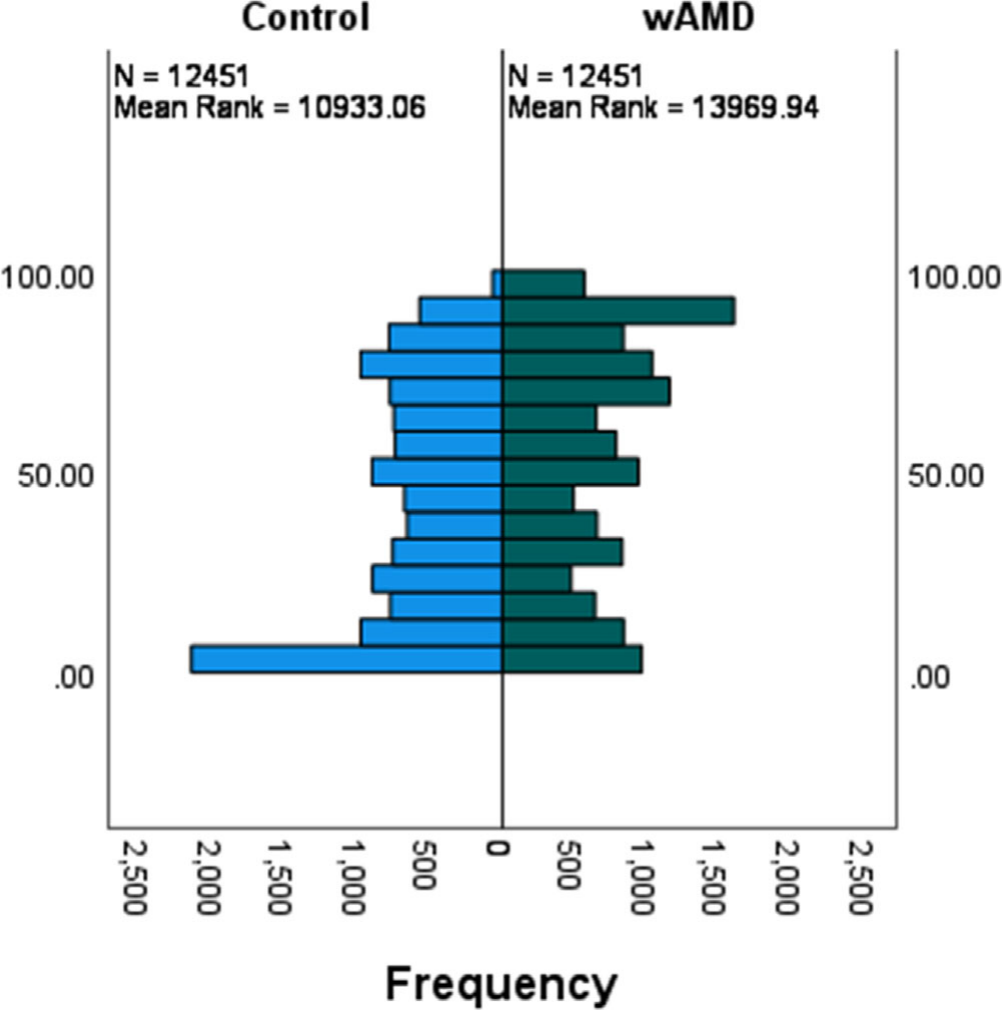
Frequency of zero counts for each filtered protein-coding gene. There were 12,451 genes left after filtering.

**Figure 3.**
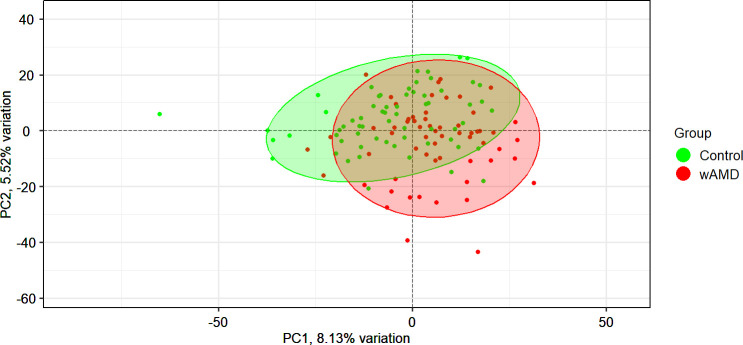
PC analysis plot of wAMD and control samples. All genes after filtering were used (12,451 in total). The ellipses cover the 95% confidence level areas of the groupings (green = controls, red = patients with wAMD).

The data contained a large number of low and zero counts. Of the potential 19,912 protein-coding RNAs, 12,451 remained after prefiltering. The patients with wAMD had zero counts for significantly more genes than the controls (median of the percentage of zero counts per gene in each group: 48.4% vs. 40.6%; *P* < 0.001), as seen in Figure 2. There was a slight separation between the groups based on a principal component (PC) analysis (Fig. 3). The PCs could not explain much of the variance in the data, with the first PC explaining 8.13%. For the first PC, the most strongly correlated patient characteristics were patient type (wAMD vs. controls; 0.35), age at sampling (0.30), anticoagulation medication use (0.22), and smoking habit (−0.27), but the correlations were fairly weak overall.

### DEGs

**Figure 4.**
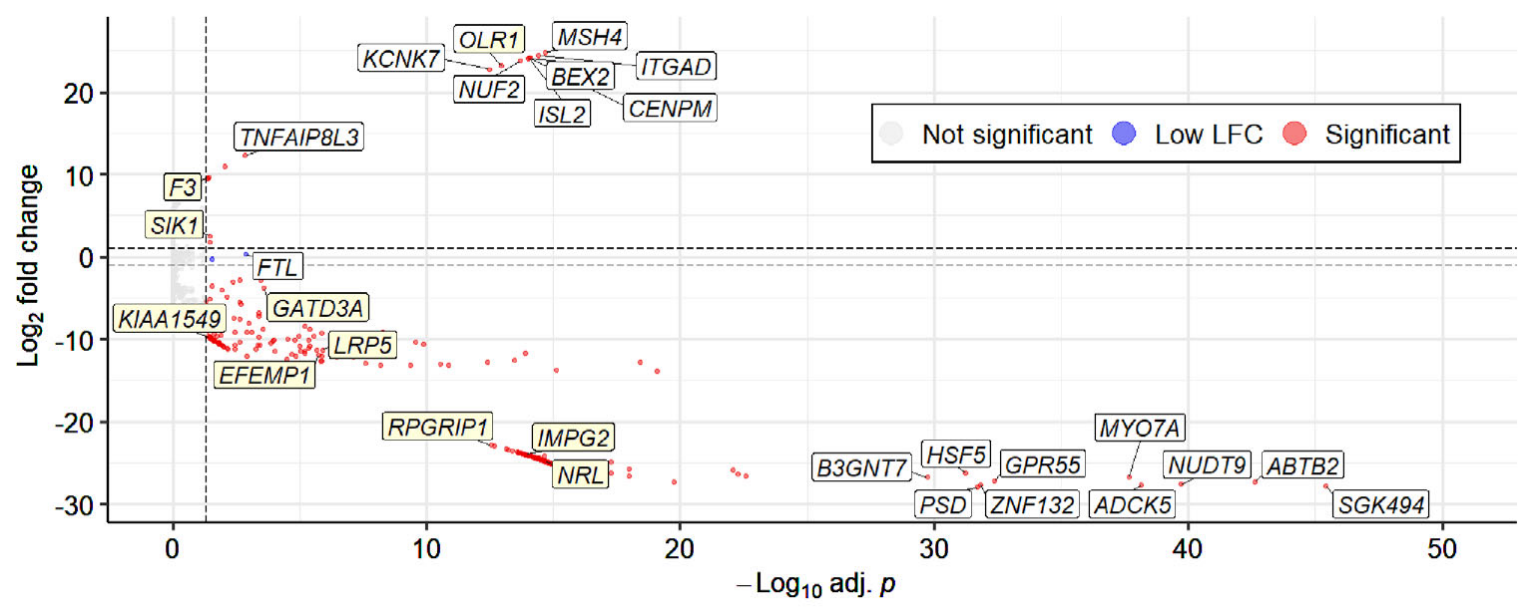
Volcano plot of the wAMD vs. control DEG results. The labeled genes represent the ten most upregulated and downregulated genes (*white label background*), as well as the genes that were found to be of interest and discussed based on later calculations and selections (*yellow label background*). DEG, differentially expressed gene; LFC, log_2_-fold change; wAMD, wet age-related macular degeneration.

There were 16 upregulated and 273 downregulated DEGs when comparing the wAMD group with the control group (Fig. 4, Supplementary Table S1). Using apeglm LFC shrinkage to adjust the results to rank genes by effect size leads to the LFCs shrinking to near-zero values, most likely due to the high proportion of low counts (Supplementary Fig. S1).

### Enriched Pathways and Terms

**Figure 5.**
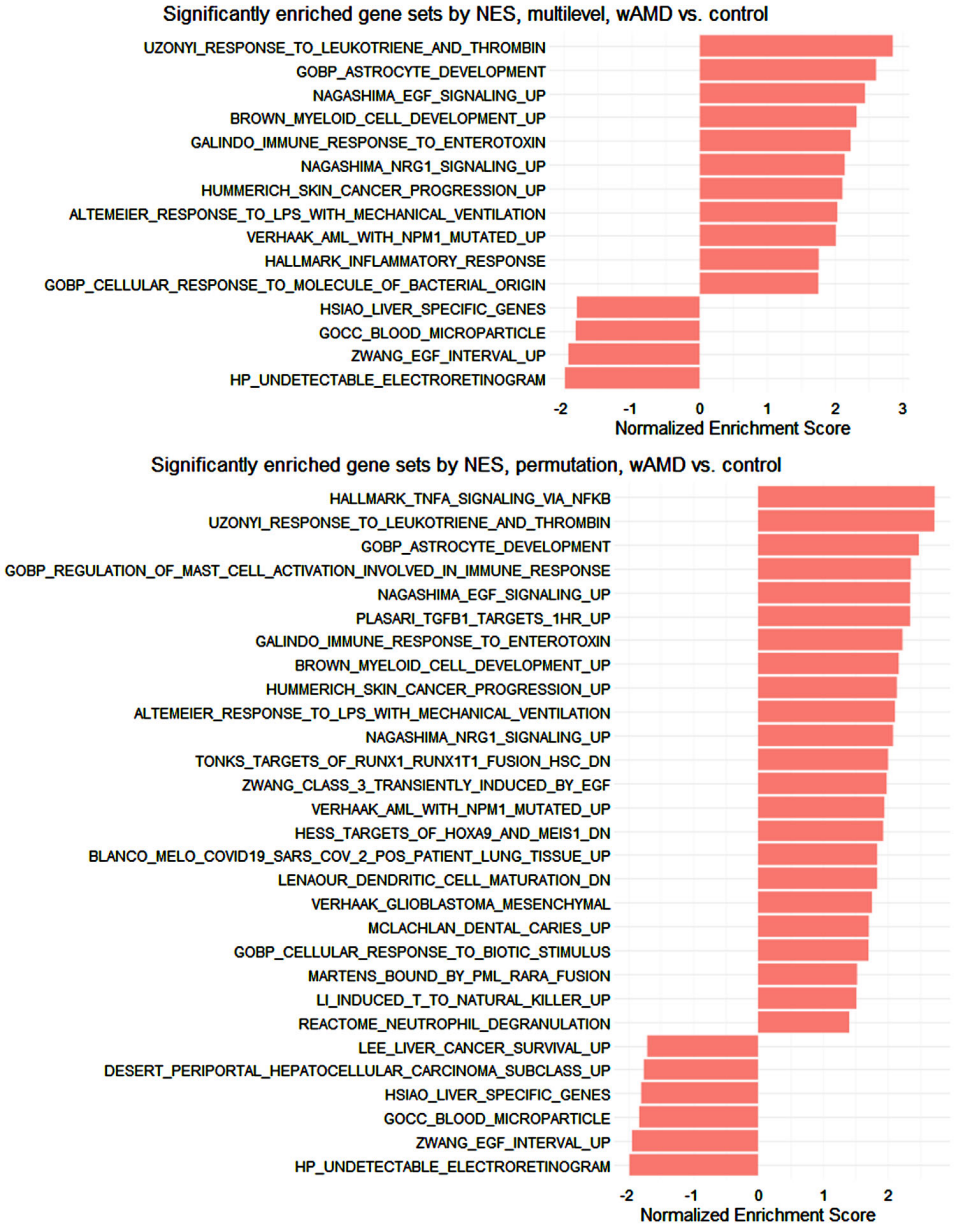
Multilevel and permutation method gene set enrichment analysis results for wAMD vs. control DEGs. Enriched gene sets with an adjusted *P* value of less than or equal to 0.050 are shown. Calculations based on the stat value (fgsea default). NES, normalized enrichment score.

The examination of the DEG results through a multilevel gene set enrichment analysis yielded 15 significantly enriched terms out of 21,802 (11 up, 4 down). As there were 91 pathways for which results could not be computed due to unbalanced (positive and negative) gene-level statistic values, another fgsea analysis was run using 1,000,000 permutations, yielding 29 significant terms (23 up, 6 down), with 3 pathways still failing to calculate. This procedure with the LFC shrunk DEGs yielded 15 enriched terms from the multilevel analysis (3 up, 12 down) and 3 downregulated ones from the million-permutation analysis. Significant gene sets for the four analyses are shown in Figure 5 and Supplementary Figure S2.

From the enriched pathways, we picked the Hallmark inflammatory response and tumour necrosis factor alpha (TNF-α) signaling via nuclear factor kappa–light chain enhancer of activated B cells (NF-κB) pathways based on inflammation being a known factor in AMD and checked which DEGs were associated with them. Three DEGs were found: oxidized low-density lipoprotein receptor 1; *OLR1* (adjusted *P* < 0.001), *SIK1* (adjusted *P* = 0.033), and coagulation factor III; *F3* (adjusted *P* = 0.042). All three were upregulated in the wAMD cohort and are associated with TNF-α signaling via NF-κB, while *OLR1* and *F3* are also associated with inflammatory response. In a gender, smoking, age at sampling, and body mass index–adjusted model, an fgsea permutation analysis also showed TNF-α signaling via NF-κB enriched, with the two associated DEGs, interleukin 6 (*IL-6*) and inducible T-cell co-stimulator ligand (*ICOSLG*), being elevated in the control cohort.

In addition to the significantly enriched pathways, we wanted to see if we had any DEGs related to retinal and macular pathological processes regardless of enrichment. Of the 57 available pathways containing the words “retina” or “macula,” an experienced clinician (K.K.) selected some that are closely related to wAMD progression. The pathways, along with the associated significant DEGs, are shown in Table 3. All of these DEGs seemed to be downregulated in patients with wAMD.

**Table 3.** DEGs From the wAMD vs. Control Analysis Related to Retinal Pathological Processes

DEG	Macular Atrophy	Macular Degeneration	Retinal Neovascularization	RPE Atrophy	RPE Mottling	LFC	*P* Value
*EFEMP1*	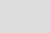	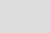			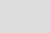	−11.93	<0.001
*IMPG2*				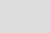		−24.02	<0.001
*KIAA1549*				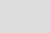		−9.76	0.035
*LRP5*			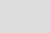			−11.26	<0.001
*NRL*	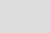	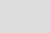				−24.81	<0.001
*RPGRIP1*		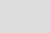				−22.86	<0.001

DEG process relationships highlighted.

*EFEMP1*, epidermal growth factor containing fibulin extracellular matrix protein 1; *IMPG2*, interphotoreceptor matrix proteoglycan 2; LFC, log2-fold change; *LRP5*, LDL receptor related protein 5; *NRL*, neural retina leucine zipper; *RPGRIP1*, retinitis pigmentosa GTPase regulator interacting protein 1.

### Links to Clinical Data

To determine the clinical relevance of the identified DEGs, we further analyzed if there were links to clinical signs of disease progression, such as retinal atrophy level and the various aspects of the anti-VEGF treatments. When patients with incomplete outer retinal atrophy (iORA) were compared with those with complete RPE and outer retinal atrophy (cRORA), none of the identified DEGs were significantly associated (atrophy status at time of sampling, data not shown). Interestingly, significantly higher *GATD3A* counts were observed in samples that were collected before initiation of anti-VEGF treatment than in samples that were taken during the treatments (*P* = 0.050; counts per million (median, 0.03 [min–max, 0.00–29.59] vs. median, 0.00 [min–max, 0.00–0.20]), treatment-naïve vs. treated patients, respectively).

### Validation

**Figure 6.**
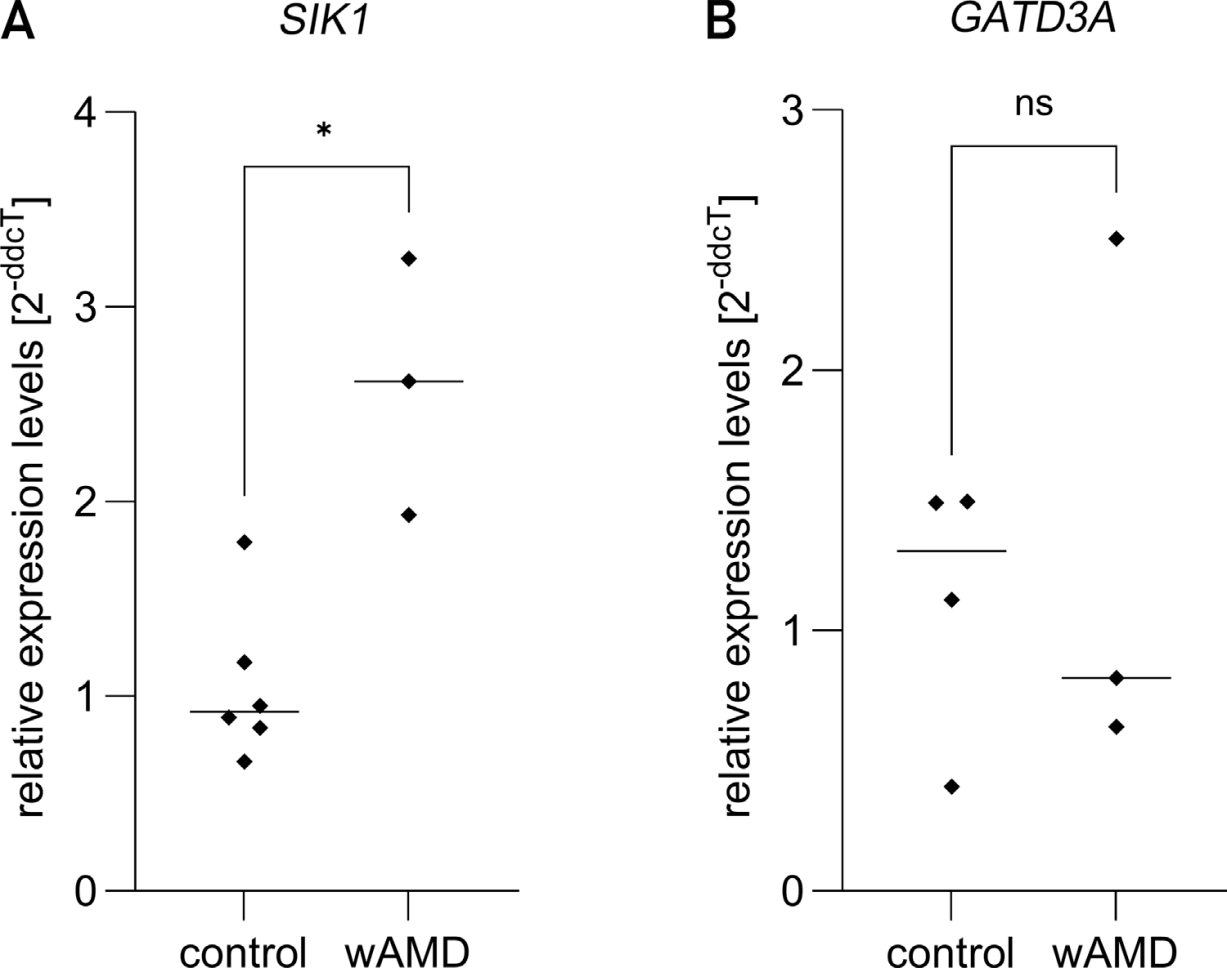
Scatter plot of the results of the qPCR analysis of (**A**) *SIK1* and (**B**) *GATD3A* mRNA expression in the serum of wAMD vs. control patients. Results are shown as a scatter dot plot with median. *GATD3A*, glutamine amidotransferase-like class 1 domain-containing 3A; *SIK1*, salt inducible kinase 1; ns, not statistically significant. *P ≤ 0.050, Mann–Whitney *U* test.

To confirm the findings, *SIK1* and *GATD3A* expression levels were reanalyzed by conventional qPCR methods. In line with the observed low and zero counts from RNA sequencing and compounded by the limitations of qPCR detection, *GATD3A* and *SIK1* could only be measured from 44% and 56% of the studied serum samples, respectively. Consequently, *GATD3A* data remained statistically nonsignificant, although the observed trend seemed to align with RNA sequencing results. *SIK1* expression levels, however, showed a 2.6-fold increase in wAMD serum samples, in agreement with the RNA sequencing data and reaching statistical significance (Fig. 6).

## Discussion

Among the Hallmark gene set-related DEGs detected in serum, *OLR1* (*LOX-1*) is expressed in neovasculature and macrophages.^50^ It acts as a receptor for oxidized low-density lipoproteins and plays an important role in the development of atherosclerosis via the activation of proinflammatory signaling pathways.^51^ Uptake of oxidized low-density lipoproteins orchestrated by OLR1 contributes to the formation of a ROS-rich environment.^52^ Along the same lines, OLR1 is present during AMD and retinal degeneration caused by neovascularization-induced inflammation, and its inhibition may have damage-reductive potential.^53^^,^^54^ SIK1 belongs to the AMP-activated protein kinase family, thus having a role in the regulation of energy metabolism within cells.^55^ It modulates glucose uptake and gluconeogenesis as well as lipid metabolism, mostly through inhibitory effects. SIK1 typically acts as a negative regulator of epithelial–mesenchymal transition. TGF-β signaling promotes SIK1 production, which in turn inhibits TGF-β and other epithelial–mesenchymal transition–related signaling pathways. SIK1, as part of the sodium-sensing network, is likely to have a part in the development of high blood pressure, which is often considered one of the many risk factors in AMD.^56^^,^^57^ Reduction of intraocular inflammation in experimental autoimmune uveitis through a mechanism involving phosphorylation of SIK1 has been proposed.^58^ SIK1 also plays an important role in circadian rhythm regulation in the retinal photoreceptors.^59^ Additionally, SIK1 has been shown to take part in hepatocyte ROS production in hyperinsulinemia-induced insulin resistance.^60^ F3, also known as tissue factor, is a blood clotting factor that plays a role in injury and immune response and is involved in the development of atherosclerotic plaques.^61^^,^^62^ It also plays a role in angiogenesis through multiple molecular pathways involving angiogenic factors such as integrins, IL-8, and VEGF.^63^ Endothelial F3 expression is regulated by early growth response 1 via the TNF-α and VEGF-activated pathways.^64^ Tissue factor is considerably increased in the retinas of patients with wAMD.^65^

MSigDB Hallmark TNF-α signaling via NF-κB is a gene set consisting of 200 genes regulated by NF-κB due to stimulus by TNF-α.^47^ NF-κB signaling can be divided into the canonical and noncanonical pathways, where the canonical pathway is usually activated via soluble TNF-α ligation to TNF receptor 1, leading to the degradation of κB inhibitor, alpha and the subsequent activation of NF-κB.^66^ Many cellular processes can be activated by this process, but the most typical ones are related to proinflammatory signaling and cell survival. Previous studies have shown that TNF-α levels are often increased in patients with wAMD, and restricting TNF-α and NF-κB activity has been shown to ameliorate inflammation and CNV within the eye.^67^^–^^70^ TNF-α, together with TGF-β, can induce epithelial–mesenchymal transition in RPE cells, which is a cellular morphology and function-altering process that has been postulated to be involved in the progression of wAMD and other ocular diseases. Inhibition of NF-κB activation via inhibitor of nuclear factor kappa-B kinase subunit beta inhibition in RPE cells under TGF-β and TNF-α stimulus has been shown to help RPE cells retain their natural characteristics.^71^ NF-κB plays an important role in the regulation of VEGF expression under adverse conditions.^72^^,^^73^ In RPE cells, stimulation by TNF-α has been shown to affect VEGF production, depending on cell polarization due to differences in NF-κB localization, which has been suggested to explain some of the differences between dry AMD and wAMD.^74^ Oxidative stress and NF-κB signaling are intrinsically intertwined as studies have shown that they are capable of suppressing and promoting each other.^75^ ROS accumulation triggers inflammatory molecule production, including that of TNF-α, which can lead to NF-κB activation, while oxidation products are capable of suppressing NF-κB activation. In contrast, NF-κB can have a protective role through routes such as autophagy promotion and prevention of TNF-α-induced apoptosis, but it can also lead to pro-oxidant molecule production. A study analyzing three human AMD transcriptome datasets from the GEO database also found the Hallmark TNF-α signaling via NF-κB to be significantly enriched, though their AMD meta-cohort consisted of dry AMD patient samples only.^76^

The response to leukotriene and thrombin gene set lists genes upregulated by thrombin (coagulation factor II, F2) and leukotriene (leukotriene D4, LTD4) in human umbilical vein endothelial cells which are commonly used to study primary endothelial cell function.^77^ Endothelial cell activation through cysteinyl leukotriene receptors and protease-activated receptor-1, to which F2 binds, leads to proinflammatory changes in vasculature, including increased IL-8 formation and secretion, a chemokine that increased in the serum of our patients in our prior study.^32^ In the present study, DEGs related to this gene set were *F3* and *SIK1*. Astrocyte development gene set focuses on the development of an astrocyte cell. In the retina and optic nerve, astrocytes protect the retinal ganglion cells from mechanical and chemical stress and regulate nutrient transport from the vasculature.^78^ There were no significant DEGs related to this gene set in our study, with vimentin coming closest to significance at adjusted *P* = 0.067. Nagashima epidermal growth factor (EGF) signaling up gene set is related to a MCF-7 cell study, where the cells were stimulated with EGF and heregulin to study their effects on MCF-7 cell morphology.^79^ *SIK1* was the only related DEG in our study.

Of the DEGs related to retinal pathological processes, LDL receptor-related protein 5 (LRP5) is part of the Wnt/β-catenin signaling pathway and therefore affects a wide-ranging array of cellular processes and pathologies. These include changes in eye vasculature and cardiovascular disease.^80^ LRP5 plays a role in macrophage function, with higher levels of LRP5 promoting anti-inflammatory macrophage subtype formation with higher migratory and phagocytic capacity.^81^^,^^82^ Within the ocular context, LRP5 mutations have been connected to retinopathies, and it has been shown to have an important role in vascular development in the retina.^83^^,^^84^ Interphotoreceptor matrix proteoglycan 2 (IMPG2) mutations are associated with retinitis pigmentosa (RP) and macular dystrophy.^85^^,^^86^ KIAA1549 is related to autosomal RP.^87^ Increased levels of EGF-containing fibulin-like extracellular matrix protein 1 (EFEMP1), also known as fibulin 3, have been shown to reduce vascular remodeling and oxidative stress in hypertension and slow down vascular smooth cell calcification.^88^ Angiotensin II has been shown to suppress *EFEMP1* expression via the NF-κB signaling pathway.^89^ EFEMP1 has also been associated with Doyne honeycomb retinal dystrophy and AMD.^90^^–^^92^ Neural retina leucine zipper is involved in rod photoreceptor cell development and maintenance and has been associated with RP.^93^^–^^95^ Last, RP GTPase regulator-interacting protein 1 (RPGRIP1) is related to degenerative diseases such as Leber congenital amaurosis, RP, and cone–rod dystrophy.^96^^–^^99^

To better understand the relevance of our findings, we explored if any of the DEGs we found were associated with disease progression. As has been established, iORA represents an early phase in retinal atrophy as only architectural thinning of the outer nuclear layer can be seen.^33^ In iORA, discontinuous loss of the ellipsoid zone has occurred and the interdigitation zone is no longer visible. In contrast, cRORA can be considered the end stage of atrophy. During cRORA, in an at least 250-µm-wide zone, the absence of RPE with associated choroidal hypertransmission together with the findings of complete outer retinal atrophy can be seen. Thus, we compared samples with iORA and cRORA, but no significant differences in the DEG counts could be found.

Extracellular vesicles can carry RNAs for long distances within the blood circulation while also keeping them relatively safe from the otherwise hostile environment.^100^^,^^101^ Although extracellular vesicles have an important role in localized retinal upkeep and are also capable of crossing the blood–retinal barrier, it is not clear how much of an effect systemic RNA factors might have on AMD development and progression.^102^ Similarly, the extent of the effect that ongoing local retinal AMD-related progresses have on the systemic circulation is not known. However, significant associations between circulating miRNAs and AMD have been detected.^26^^,^^103^^,^^104^ The blood-retinal barrier weakens due to aging and becomes leaky as CNV progresses, so the role systemic factors play may increase as the disease progresses.^105^^,^^106^

Treatment with intravitreal injections of anti-VEGF agents is the standard therapy for wAMD. Anti-VEGF agents act by preventing neovascularization and normalizing the function of existing vessels. Thus, they decrease fluid leakage from newly formed or altered capillaries as seen in CNV. As treatment with anti-VEGF agents is considered to slow down disease progression and weaken active disease, we explored whether treatment with anti-VEGFs can modify serum mRNA levels for the genes of our interest. Indeed, levels of *GATD3A* were observed to decrease during anti-VEGF treatment. The *GATD3A* levels were higher in controls than in wAMD samples, so the observed difference may result from modulation by anti-VEGF agents or disease progression during the treatment. The function of *GATD3A* during AMD development is unknown, but Smith et al.^107^ have recently reported that *GATD3A* acts as a deglycase in the mitochondrial matrix. It is well-established that the number and function of mitochondria are reduced in AMD, which may present a potential linkage to our finding.^16^

The effectiveness of treatments is not the same in every patient and can decrease with time even in those who initially respond well to treatment. For example, in wAMD, the effectiveness of the intravitreal anti-VEGF treatments is often found to drop off after 3 or so years, and fibrotic changes in the retina brought on by the accumulating damage have been suggested to at least partially explain the reduction in the effectiveness of the anti-VEGF injections.^108^^,^^109^ TNF-α and NF-κB have been shown to modulate fibrotic events in multiple organs, including the eye, both via direct and indirect regulation of fibrogenic events and through inflammatory signaling pathways.^110^^–^^113^

**Figure 7.**
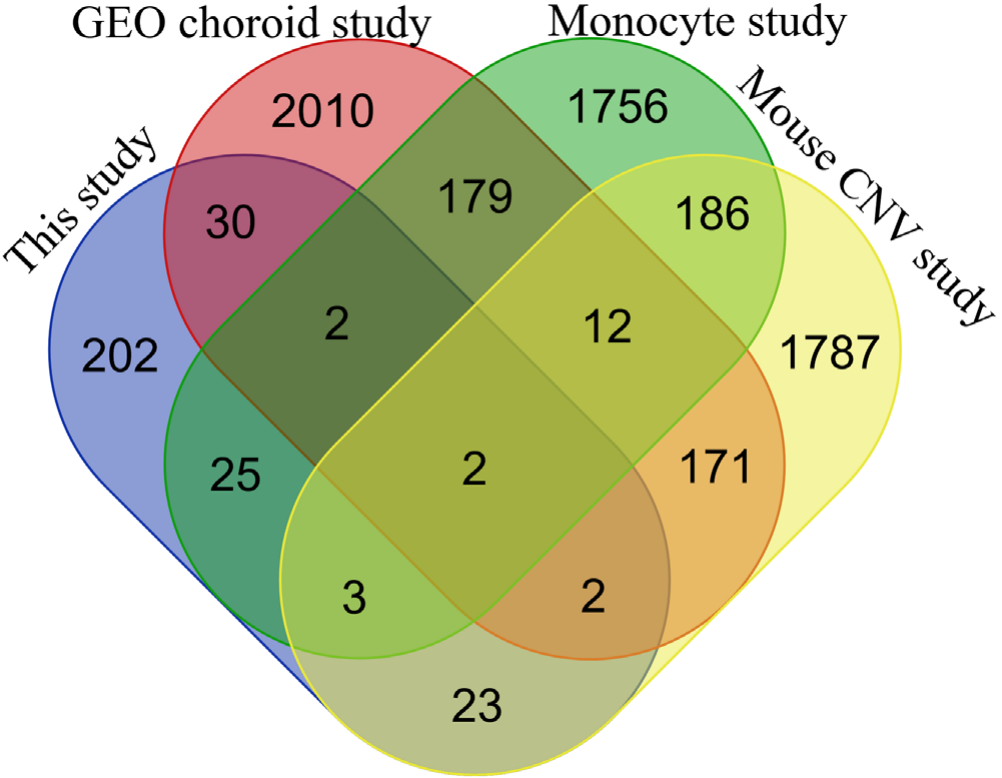
Venn diagram of the overlap of DEGs in our and three other studies related to AMD research.

To see if our DEG list has significant similarities to prior studies, we compared our DESeq2 results with some publicly available transcriptomic datasets created by AMD-related studies. In an RPE choroidal tissue study based on GEO database data, 2048 DEGs were found using the limma package on R.^114^ Of these DEGs, 36 were the same as ours. From their further filtered list of 50 genes, one overlapped with ours (nuclear pore complex interacting protein family member B5, *NPIPB5*). A monocyte study found 2165 DEGs through robust multiarray average analysis and ANOVA, of which 32 overlapped with ours. From their further filtered list of 79 genes, 1 overlapped with ours (*OLR1*).^115^ In a laser-induced CNV study with mice, 2186 DEGs were found from RPE–choroidal samples using EdgeR, of which 30 overlapped with ours, including *SIK1*.^116^ In short, roughly 11% of our study's DEGs overlap with the abovementioned studies’, whereas each of the three studies’ DEGs overlap with each other by roughly 9% (Fig. 7). None of these overlaps are greater than what would be expected from random sampling based on hypergeometric distribution testing. Differences arising from study demographics, sample types, and testing methodologies make it rather difficult to draw conclusions from similarities, or lack thereof, to prior studies, especially in such a constantly evolving field such as RNA research.^117^ We believe that our study is useful as a data source upon which future serum-based mRNA studies would be able to reflect their results on. Thus far, AMD studies on serum mRNA have mostly concentrated on singular transcripts, so we would be interested in seeing more similar types of studies as ours be conducted.

Our study has several limitations. The RNA concentrations in the samples were rather low, but the sequencing data yield was considered fine. A considerable proportion of low and zero counts in the samples can lead to exaggerated differences in the results, low effect sizes, and difficulties in interpretation. Also, the poor ability of the PCs to explain the variance in the data may indicate the existence of unknown factors influencing the findings. Aqueous humor samples could not be taken due to ethical restrictions, preventing their comparison with serum samples. An additional hurdle is brought on by the variation between prior studies, which also leads to difficulties in result interpretation. In contrast, these issues could, at least in part, be seen as rather natural as real-world data was used. Data from conventional qPCR supported our findings for *SIK1* and *GATD3A*, although low sensitivity and limited sample numbers decreased the statistical power of these findings. Still, successful replication of our findings with RNA sequencing by qPCR supports our findings.

In summary, protein-coding RNAs of inflammation-related pathways are enriched in Finnish patients with wAMD compared with a control cohort. Within these pathways, TNF-α signaling via NF-κB is of particular interest due to its relationship with inflammation, mitochondrial energy metabolism, VEGF regulation, and fibrosis, which are important factors in the neovascularization process and treatment of wAMD. The levels of serum *GATD3A* RNA were found to be significantly lower in patients who had been receiving anti-VEGF treatments before sampling. Many of the DEGs also had connections to cardiovascular diseases, which are risk factors for AMD. Blood serum analysis provides systemic insight into wAMD, which might help to explain the mechanisms of disease progression. Gathering even larger amounts of data could lead to more robust findings that might further help develop a better understanding of the etiology of wAMD.

## Supplementary Material

Supplement 1

Supplement 2
