# Supplementary material for: Oxidative Stress and Inflammation-Related mRNAs Are Elevated in Serum of a Finnish Wet AMD Cohort
Source: Invest Ophthalmol Vis Sci. 2024 Nov 15;65(13):30. doi: 10.1167/iovs.65.13.30 (PMC11578155; doi:10.1167/iovs.65.13.30)
Supplement: Supplement 1 [file iovs-65-13-30_s001.pdf]

Supplementary Material

Table S1. List of the up- and downregulated DEGs, wAMD vs. control.
Separate file
The mean count represents the average of the normalized count values, divided by size factors, taken over all samples.
DEG = differentially expressed gene, LFC = $\log_2$-fold change, wAMD = wet age-related macular degeneration

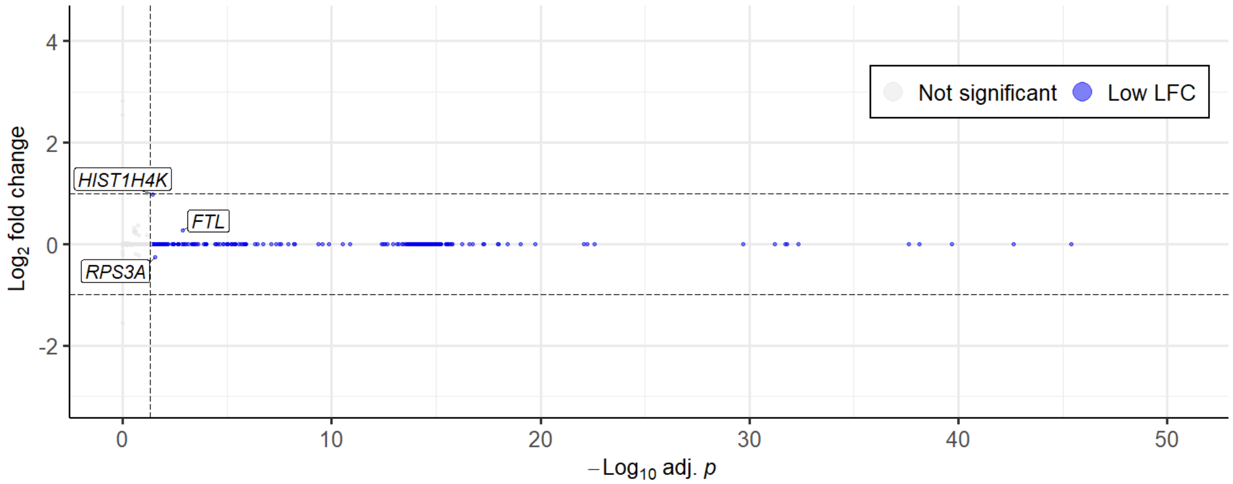

Figure S1. Volcano plot of the wAMD vs. control DEG results with apeglm LFC shrinkage. Named genes represent every significant gene with > 0.1 adjusted LFC in either direction. DEG = differentially expressed gene, LFC = $\log_2$-fold change, wAMD = wet age-related macular degeneration

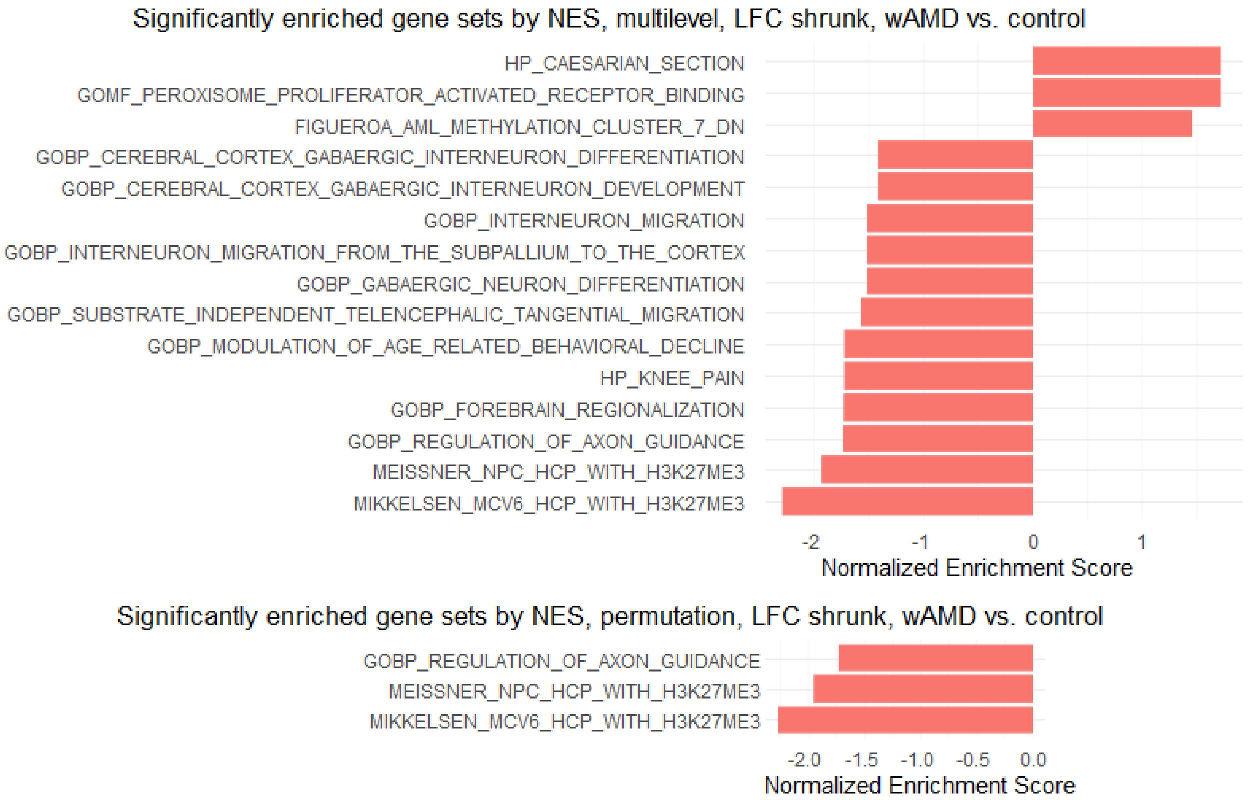

Figure S2. Multilevel and permutation method gene set enrichment analysis results for LFC shrunk wAMD vs. control DEGs. Enriched gene sets with an adjusted *p*-value of < 0.05 shown. No incomputable pathways were found in either fgsea run. Results are based on the $\log_2$-fold change value (DESeq2 recommendation, as the stat values are not preserved in the data transformation). DEG = differentially expressed gene, LFC = $\log_2$-fold change, NES = normalized enrichment score, wAMD = wet age-related macular degeneration
